# EEG-Parameter-Guided Anesthesia for Prevention of Emergence Delirium in Children

**DOI:** 10.3390/brainsci12091195

**Published:** 2022-09-05

**Authors:** Yaqian Han, Mengrong Miao, Pule Li, Yitian Yang, Hui Zhang, Beibei Zhang, Mingyang Sun, Jiaqiang Zhang

**Affiliations:** 1 Department of Anesthesiology and Perioperative Medicine, Henan Provincial People’s Hospital, People’s Hospital of Zhengzhou University, Zhengzhou 450003, China; 2 Department of Anesthesiology and Perioperative Medicine, People’s Hospital of Zhengzhou University, Zhengzhou 450003, China; 3 Department of Anesthesiology, Tengzhou Central People’s Hospital, Jining Medical College, Tengzhou 277522, China

**Keywords:** emergence delirium, pediatric anesthesia, anesthetic depth, SEF, DSA, EEG

## Abstract

Background: Emergence delirium (ED) usually occurs in children after surgery with an incidence of 10−80%. Though ED is mostly self-limited, its potential injuries cannot be ignored. Whether electroencephalography (EEG)-parameter-guided anesthesia could reduce the incidence of ED in pediatric surgery has not been fully discussed to date. Methods: Fifty-four boys aged 2–12 years undergoing elective hypospadias surgery under sevoflurane anesthesia were selected. In the EEG-parameter-guided group (E group), sevoflurane was used for anesthesia induction and was maintained by titrating the spectral edge frequency (SEF) to 10–15 and combining the monitoring of density spectral array (DSA) power spectra and raw EEG. While in the control group (C group), anesthesiologists were blinded to the SedLine screen (including SEF, DSA, and raw EEG) and adjusted the intraoperative drug usage according to their experience. Patients with a Pediatric Anesthesia Emergence Delirium (PAED) score > 10 were diagnosed with ED, while patients with a PAED score > 2 were diagnosed with emergence agitation (EA). Results: Finally, a total of 37 patients were included in this trial. The incidence of ED in the E group was lower than in the C group (5.6% vs. 36.8%; *p* = 0.04), while the incidence of EA was similar in the two groups (61% vs. 78.9%; *p* = 0.48). Intraoperative parameters including remifentanil dosage and the decrease in mean arterial pressure (MAP) were not different between the two groups (*p* > 0.05), but the mean end-tidal sevoflurane concentration (EtSevo) was lower in the E group than in the C group (*p* > 0.05). Moreover, during PACU stay, the extubation time and discharge time of the groups were similar, while the PAED scores within 5 min from extubation and the Face, Legs, Activity, Cry, and Consolability (FLACC) scores within 30 min from extubation were lower in the E group than in the C group. Conclusion: EEG-parameter-guided anesthesia management reduced the incidence of ED in children. Studies with larger sample sizes are needed to obtain more convincing results.

## 1. Introduction

ED is an acute postoperative behavioral change that is defined as a disturbance in a child’s awareness or attention to their environment, usually accompanied by disorientation and perceptual alterations, including hypersensitivity to stimuli and hyperactive motor behavior in the immediate post-anesthesia period [1]. Its incidence ranges from 10 to 80% in preschool children who receive sevoflurane anesthesia [2]. Though ED is mostly self-limited and occurs within about 5–15 min during the emergence period [3], its physical injuries cannot be ignored, as children may wiggle the limbs, get rid of their catheters, and even get out of important equipment during their treatment [4].

The prevention of ED includes pharmacological treatment and non-pharmacological treatment. Pharmacological treatment involving the administration of midazolam, ketamine, dexmedetomidine, and melatonin during the preoperative or intraoperative period is effective, but these measures may prolong the stay in the postoperative anesthesia care unit (PACU) and cause many adverse reactions, such as postoperative nausea and vomiting, respiratory depression, and others [5,6,7,8,9]. Non-pharmacological treatment, such as parental companionship, preoperative education, or playing music after entering the room, present therapeutic effects for lower cost and more convenience [2,10,11]. So, it is valuable to find ways to prevent ED using non-pharmacological treatments. 

At present, more and more anesthesiologists titrate anesthesia dosage by monitoring the depth of anesthesia [12,13,14]. In 2020, EEG monitoring was recommended as one of the important organ monitoring methods for guiding general anesthesia management by the American Society of Anesthesiologists (ASA) [15]. In the adult population, the potential benefits of the intraoperative monitoring of anesthesia depth, including a lower incidence of hypotension under anesthesia and intraoperative awareness, faster awakening and recovery time, and less drug dosage use, were confirmed [16,17]. Although the effect of bispectral index (BIS) monitoring on preventing postoperative delirium (POD) remains controversial [17,18], multiple meta-analyses showed that anesthesia management via EEG monitoring could reduce the occurrence of POD in adult patients undergoing general anesthesia [19,20]. Monitoring EEG and anesthetic depth has been used in pediatric anesthesia management since 2000 [21]; then, it was suggested to be used in children who undergo major or long-term surgery [22,23]. It was proved that EEG monitoring in pediatric anesthesia is beneficial for children [12,14,24]. However, to date, pediatric routine anesthesia management does not include anesthetic depth monitoring but is instead largely dependent on the anesthesiologist’s experience.

At present, the correlation between ED and anesthetic depth in previous studies mainly focused on numeric proprietary indices such as the BIS (Medtronic, Minnesota, Minnesota), state entropy (SE), response entropy (RE), and the Narcotrend index (MonitorTechnik, Bad Bramstedt, Germany) [24,25,26,27]. As the calculation algorithms of these monitoring indices are based on adult EEG characteristics, the current indices may not suitable for children undergoing general anesthesia [27,28,29].

Recently, Xu et al. reported that the SEF, DSA, and raw EEG could be more effective for reflecting anesthetic depth than proprietary indices (e.g., BIS, Narcotrend index, and Patient State Index (PSI)) in pediatric surgery [12,30]. Other studies also reported that the SEF could be more effective in representing anesthetic depth [31,32]. Moreover, an observational study also found that the DSA could be available as a measure of anesthetic depth in young children undergoing sevoflurane anesthesia [33]. Additionally, Koch et al.’s trials analyzed raw EEG characteristics in children undergoing general anesthesia and found some relationships between ED and EEG epileptiform discharges [34]. However, whether using these EEG parameters (SEF, DSA, raw EEG) could reduce the incidence of ED is still uncertain.

In this work, we used the SEF, DSA, and raw EEG, but not the BIS, to monitor anesthetic depth in children because: (1) proprietary quantitative indices such as the BIS may not be well suited for children [26,27] and (2) the SEF, DSA, and raw EEG may more precisely reflect anesthetic depth. Our aim was to explore whether using the SEF, DSA, and raw EEG to guide pediatric anesthesia could reduce the incidence of ED.

## 2. Methods

### 2.1. Ethical Statement

The study was approved by the Research Ethics Committee of Henan Provincial People’s Hospital ((2021) 129), Henan, China, and was prospectively registered in the Chinese Clinical Trial Registry (ChiCTR2100050263) on 5 September 2021. All subjects provided written informed consent, signed by their parents and/or themselves.

### 2.2. Participants

Patients admitted to Henan Provincial People’s Hospital were recruited from 15 October 2021 to 15 February 2022. The inclusion criteria were (1) aged 2–12 years, (2) ASA grades I and II, (3) no history of serious neurological or mental illness, (4) elective hypospadias operation under general anesthesia, and (5) length of anesthesia expected to be more than 1 h. The exclusion criteria were (1) patients with any history of neurological or mental illness and any signs of growth retardation, (2) parents and subjects were unable to cooperate or participated in another research study at the same time.

### 2.3. Randomization and Blinding

**Figure 1:**
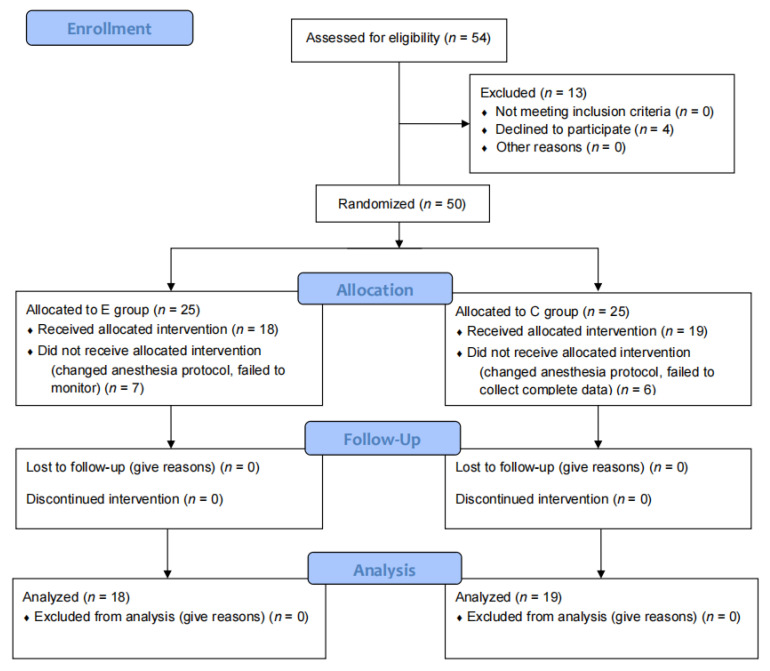
CONSORT flowchart of recruitment and inclusion of subjects.

Informed consent was signed before the day of surgery and patients were randomly assigned (1:1) to a control group (C group; anesthesiologists depended on their experience to manage anesthesia) or a EEG-guided group (E group; anesthesiologists depended on the SEF, DSA, and raw EEG to guide anesthetic depth) using the random number method in SPSS 25.0. Participants, follow-up staff in preoperative room or in the PACU, and the EEG-analysis physician were blinded to allocation. A total of 54 patients were enrolled, and 37 patients were finally divided into two groups, with 19 participants (19 males; mean age of 4.79 ± 2.92 years) in the C group and 18 participants (19 males; mean age of 5.67 ± 2.77 years) in the E group (Figure 1). Table 1 lists their demographic and baseline characteristics.

### 2.4. Study Protocol

All participants’ baseline characteristics were collected before the day of surgery, and the legal guardians of patients were contacted by the researchers and signed informed consent (CONSORT flowchart; see Figure 1). All patients were prepared with intravenous access one day before surgery. 

On the day of surgery, in the preoperative room, 0.05 mg/kg midazolam was administered to participants 30 min before surgery, with the monitoring of oxygen saturation using a detector being performed at the same moment. The researcher who was blinded to allocation was responsible for taking care of the child in the preparation room and kept monitoring the blood oxygen saturation (SPO_2_) and the heart rate (HR). If there was a bradycardic heart rate or respiratory depression, such as a significant decrease in blood oxygen saturation or a decrease in the frequency of breaths, mask inhalation of oxygen was immediately arranged.

#### 2.4.1. EEG Monitoring

**Figure 2:**
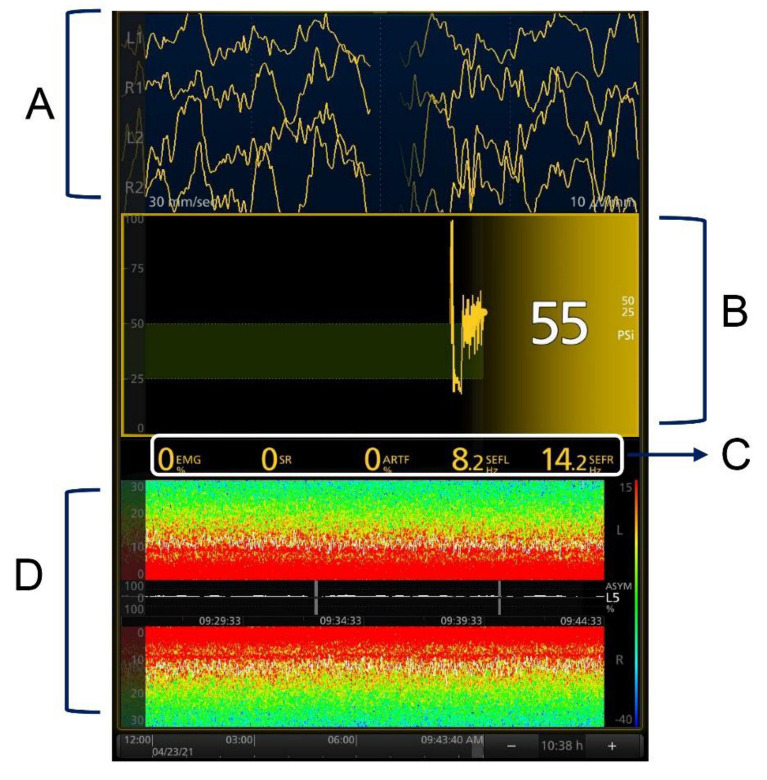
Interpretation of SedLine Monitor indices. (**A**) The four channels of EEG from top to bottom are, respectively, L1 (FP1), R1 (FP2), L2 (F7), and R2 (F8) synchronous electrical activities. (**B**) PSI, specific quantitative index of anesthesia depth. (**C**) Electromyography (EMG), which represents the measurement index of temporal muscle relaxation. SR, burst suppression; the presence of burst suppression indicates too-deep anesthesia. ARTF, percentage of false difference (interference). SEFL, SEFR; the distribution represents the spectral edge frequency of the left and right sides of the brain, which is the frequency range where 95% of the brain electrical activity is located. (**D**) DSA (density spectral array), which reflects the relationship between EEG power and frequency over time. The white line represents the spectral marginal rate including the SEF; the upper and lower parts are the spectral arrays of the left and right sides of the brain; and the color band on the right represents the power intensity. The higher the power, the brighter the spectral array in the frequency range where it is located.

**Figure 3:**
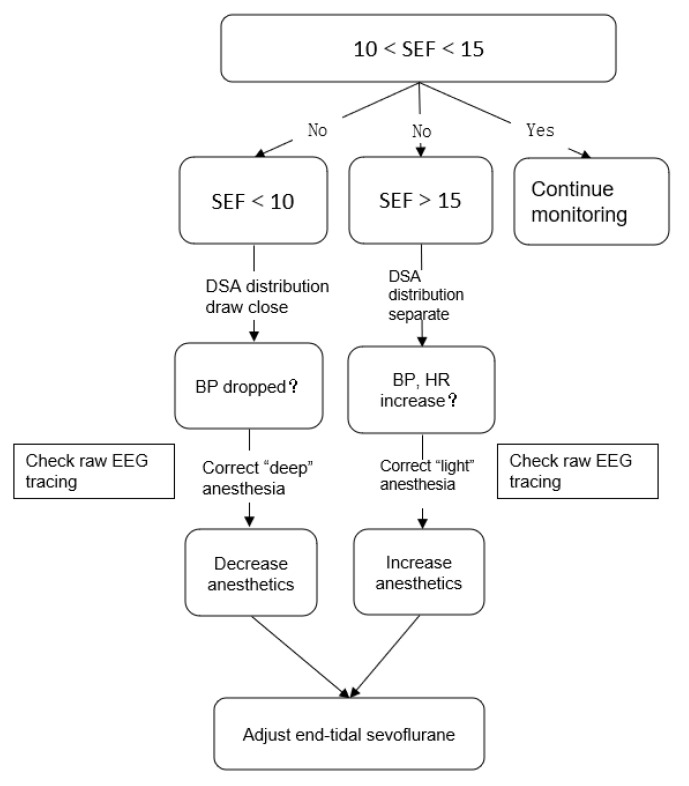
Intervention flow diagram. SEF, spectral edge frequency; BP, blood pressure; HR, heart rate; EEG, electroencephalography.

After entering the operating room, participants were connected with routine monitoring and SedLine EEG Monitor (Masimo, Irvine, CA, USA). The electrodes were placed in FP1, FP2, F7, and F8. The specific meaning of each index is shown in Figure 2. The recommended SEF range for the maintenance of general anesthesia is 10–15 in children [12,30,35]. The EtSevo should be immediately changed when SEF < 10 and DSA images show burst suppression (black vertical segment) and main power frequency band (red horizontal band) depression; when the EEG waveform presents burst suppression, and isoelectric and other conditions, indicating that the depth of anesthesia may be abnormal; or when the vital signs of the child show serious fluctuations. The specific adjustment method for sevoflurane is shown in Figure 3.

#### 2.4.2. Anesthesia

For anesthesia induction, the inhaling of 8% sevoflurane and the intravenous giving of 0.1–0.5 ug/kg sufentanil and 0.5 mg/kg cisatracurium were used before intubation. For anesthesia maintenance,2–4% sevoflurane and 0.02–0.2 ug/kg/min remifentanil were administered. All patients received 0.1 ug/kg sufentanil and 0.1 mg/kg tropisetron at the end of the procedure. In the E group, anesthesiologists performed anesthesia administration according to EEG-related parameters such as the SEF, DSA, and raw EEG waves. In the C group, sevoflurane was used for routine induction using the tidal volume method according to the anesthesiologists’ experience. During the whole process of anesthesia, a whiteboard was used to shelter the screen of the SedLine monitor, and the abnormal alarm of anesthesia depth index of the anesthesia depth monitor was muted (such as that for too-deep or -light anesthesia). The anesthesiologists did not know the changes in EEG and the index of anesthesia depth during the whole surgery. During the whole process, sevoflurane concentration was adjusted according to clinical experience and vital signs in this group. In both groups, the concentration of sevoflurane was reduced to 4% after the child lost consciousness. According to their spontaneous breathing, spontaneous breathing or ventilator-assisted breathing was chosen. At the end of induction, 0.5 mg/kg cis-atracurium and 0.1–0.5 ug/kg sufentanil were given. After satisfactory muscle relaxation, an endotracheal or a laryngeal mask was intubated according to the surgical needs or the patient’s condition.

During the induction of anesthesia, the EtSevo was recorded every 15 s. The vital signs of the child, including blood pressure, heart rate, and oxygen saturation, were recorded every minute.

Anesthesia maintenance was achieved with 2–4% sevoflurane and 0.02–0.2 ug/kg/min remifentanil. In group E, the anesthesiologists adjusted the EtSevo according to EEG-related parameters and raw EEG waves, and the rate of remifentanil was adjusted according to the child’s blood pressure, heart rate, and response to skin incision. The SEF, SR, and EMG values were continuously recorded every 2 s by the SedLine machine and exported as Excel files for subsequent analyses. All these data from the two groups were saved offline after removing the SedLine electrodes. The EtSevo and MAP were recorded every 5 min. The total infusion dosage of remifentanil was recorded at the end of surgery. 

Sufentanil and tropisetron at the concentrations of 0.1 ug/kg and 0.1 mg/kg, respectively, were routinely given 15–30 min before the end of surgery as an antiemetic and an analgesic. The concentration of sevoflurane was gradually reduced to 0%. Remifentanil was discontinued at the end of surgery. Subsequently, a patient-controlled intravenous analgesia (PCIA) pump was connected with patients. PCIA included 1 ug/kg^+^ sufentanil, 0.1 mg/kg^+^ tropisetron, and normal saline, for a total volume of 100 mL. All patients received continuous infusion (basal rate of 2 mL/h) and 2 mL on-demand bolus with a lockout interval of 15 min. Patients were transported to the PACU postoperatively, and the SedLine electrodes were removed before awakening to avoid disturbing patients‘ sleep.

#### 2.4.3. Clinical Scales

For all children included in this study, we used the PAED scale, which can evaluate delirium and agitation in children during the emergence period from six aspects, such as eye contact with caregivers or parents, purposeful action, awareness of the surrounding environment, restlessness, and inconsolability. A score of 1 is quiet cooperation; a score of 2 is mild discomfort—patient cries but can be soothed; a score of 3 indicates that a patient cries but cannot be soothed; a score of 4 is fidgety hands and feet, completely out of control. The higher the score, the more severe the child’s agitation; scores > 2 indicate EA, and scores > 10 indicate ED.

In this study, the FLACC scale was used to evaluate the postoperative pain of children. It comprehensively evaluates the pain degree of children from five perspectives: facial expression, leg activity, body position, crying, and comfort degree. Score standard: 0 points indicate relaxed and comfortable; 1–3 are classified as mild discomfort; 4 to 6 indicate moderate pain; 7 to 10 indicate severe pain, discomfort, or both.

Extubation was assessed by a professional anesthesiologist. We recorded the time of extubating, agitation (PAED scores), and pain (FLACC) scores at extubation and 5, 10, 15, 20, and 30 min after extubating. PAED scores > 10 were considered ED, and EA was diagnosed when PAED score > 2 [36,37]. Sufentanil at a concentration of 0.1 ug/kg was given when FLACC > 4. The FLACC score was evaluated again after 5 min, and if the score was more than 4, sufentanil could be given repeatedly until the pain score was <4.

### 2.5. Outcome Measures

The primary outcome was the incidence of ED in the PACU. The secondary outcomes during surgery were the mean EtSevo, MAP decrease extent (the maximum decrease in blood pressure compared with the baseline), and intraoperative remifentanil dosage. As for the outcomes in the PACU, the incidence of EA was studied. PAED and FLACC scores at extubation and 5, 10, 20, and 30 min after extubating, emergence time, and discharge time were also recorded.

### 2.6. Sample Size and Statistical Analysis

The reported rate of ED in similarly treated control groups is 53% [38]; assuming that the incidence of ED in the E group would decrease to 10%, the analysis using PASS 15.0 (NCSS, LLC. Kaysville, UT, USA) with a statistical power of 80% and α = 0.05 for two-tailed tests yielded a sample size of 36. Assuming a drop-out rate of 20%, 46 patients were expected to be recruited.

The chi-square test was used for the categorical variables. Continuous variables were compared using the independent-samples *t*-test or Mann–Whitney U test and were presented as means ± standard deviations or medians (Q1 and Q3). The statistical analyses were performed using SPSS software version 26.0 (IBM Corp., Armonk, NY, USA, 2019). A two-sided *p*-value of less than 0.05 was considered statistically significant. 

## 3. Results

Fifty-four participants were recruited from October 2021 to January 2022, but four of them refused to participate. Then, fifty children and their care-givers were randomly approached for participation in the study; seven in the E group and six in the C group were lost because of the change in the anesthesia protocol or the failure of monitoring. Therefore, 37 male children aged 2–11 years inclusively were finally included and assigned to two treatments (18 in the E group and 19 in the C group). The random assignment of all subjects is depicted in Figure 1.

### 3.1. Baseline Characteristics

The baseline characteristics of our trials are shown in Table 1. There were no significant differences in the two groups with respect to age, length of anesthesia, history of surgery, or intubation type.

### 3.2. Outcomes in PACU Period

The postoperative outcomes in the PACU are presented in Table 2. For the primary outcome, the incidence of ED in the E group was significantly lower than that in the C group (C group, 36.8%; E group, 5.6%; *p* = 0.042), while the incidence of EA was not statistically different between the two groups (C group, 78.9%; E group, 61.1%; *p* = 0.476). Moreover, extubation time, discharge time, and the incidence of adverse events were similar between the two groups.

**Figure 4:**
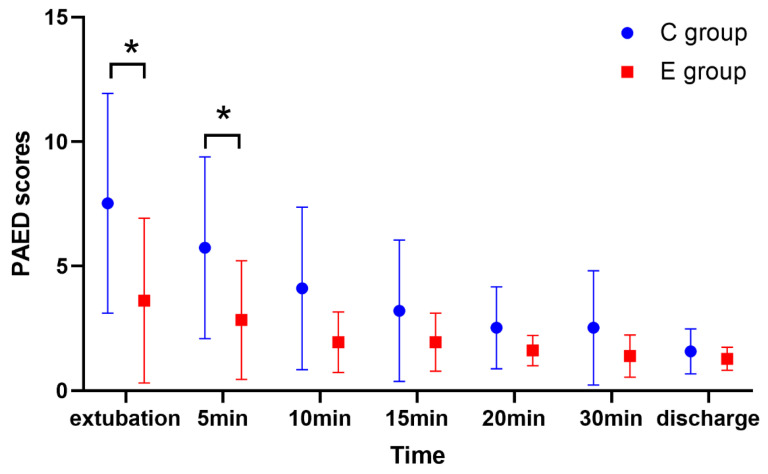
PAED scores at different time points. PAED, Pediatric Anesthesia Emergence Delirium; * *p* < 0.05.

The PAED scores were significantly lower in the E group than in the C group at extubation and 5 min after extubation (Figure 4). While the PAED scores at 10, 15, 20, and 30 min after extubation were similar between the two groups.

**Figure 5:**
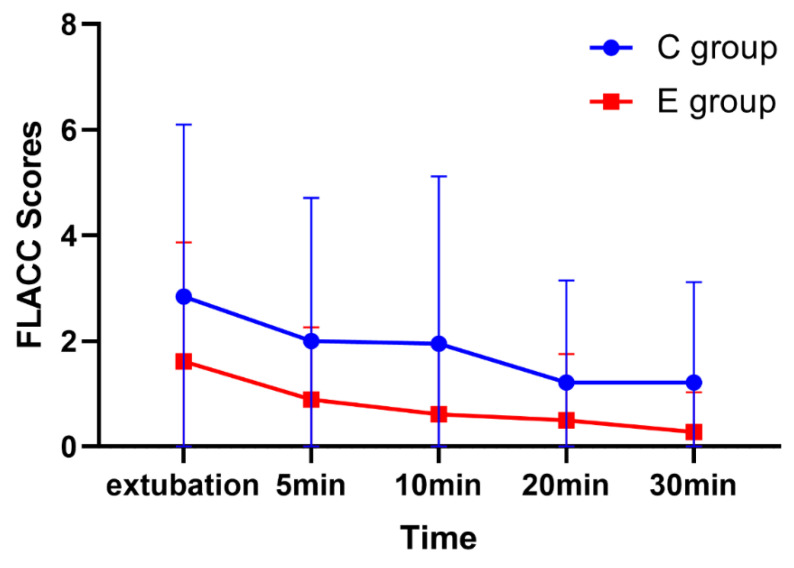
FLACC scores at different time points. FLACC, Faces, Legs, Activity, Cry, and Consolability.

As for the highest FLACC score in the PACU, the C group had a greater score than the E group within 30 min from extubation (C group, 2 (1,3); E group, 0 (0, 2); *p* = 0.032; Figure 5).

### 3.3. Intraoperative-Related Characteristics

The intraoperative characteristics are presented in Table 3. The end-tidal sevoflurane concentration during induction was similar between the two groups (5.8 ± 1.6 vs. 5.6 ± 1.9; *p* = 0.476), while the end-tidal sevoflurane concentration in the E group during maintenance was lower than that in the C group (2.8 ± 0.7 vs. 2.3 ± 0.9; *p* = 0.033). The intraoperative MAP percentage declined, and the mean remifentanil dosage showed no differences between the two groups.

**Figure 6:**
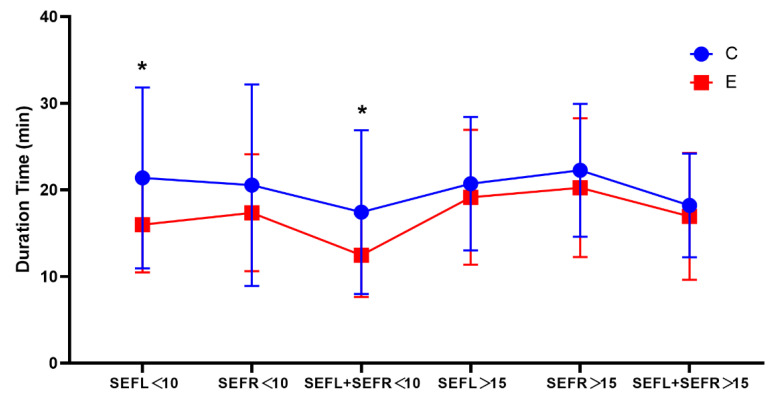
Duration of SEF < 10 or SEF > 15. SEF, spectral edge frequency; SEFL, SEF of left brain; SEFR, SEF of right brain; SEFL + SEFR, figure simultaneously representing SEFL and SEFR < 10 or >15; * *p* < 0.05.

The duration of SEF < 10 or >15 is shown in Figure 6 and Table 4. The durations of SEF left < 10 and SEF left + SEF right < 10 in the E group were significantly shorter than in the C group (15.8 ± 5.6 vs. 21.3 ± 10.1 (*p* = 0.049); 12.1 ± 4.6 vs. 17.6 ± 9.2 (*p* = 0.028)). The time of SEF > 15 was similar in both groups.

## 4. Discussion

Pediatric emergence delirium is one of the main problems after pediatric surgery; it usually occurs in the first 30 min after awakening and may cause harm to the children themselves and increase the burden of medical staff and parents [39,40]. How to minimize the occurrence of emergence delirium needs to be considered by clinicians. At present, relevant literature studies have proposed a variety of prevention methods for emergence delirium, among which there is non-drug prevention including parental companionship, preoperative education, or playing music after entering the room [41,42,43]. Additionally, delirium in intensive care unit (ICU) patients could also be prevented via non-pharmacological nursing interventions, including patient’s family, patient-centered care, and light therapy or vitamin B1 preventive treatment [44,45]. Currently, plenty of researchers have explored if it is reliable to use BIS monitoring in pediatric anesthesia; it is still controversial, because the brain development of children is not mature and there are no exact correlations between BIS values and the EtSevo [46,47,48]. In this study, intraoperative EEG parameters (SEF, DSA, and raw EEG) were used to guide anesthesia management for preventing emergence delirium, which may offer a reference value for optimizing anesthesia management in pediatric surgery.

Sullivan’s trial found that in 54 children aged 2–12 undergoing orthopedic surgery, the BIS-monitoring group (maintaining BIS values of 40–60) reduced sevoflurane exposure compared with the normal group, but the difference was not statistically significant [49]. In our trial, however, the intraoperative EtSevo was significantly reduced in the E group. The reason why these results differed from Sullivan’s trial results may be the differences in the index chosen to judge anesthesia depth; our choices were the SEF, DSA, and raw EEG instead of the quantitative index of anesthesia depth, PSI, which can be more accurate in pediatric anesthesia [30,33,47,50]. In addition, another study reported that BIS monitoring for the guidance of propofol–remifentanil anesthesia did not result in a reduced consumption of remifentanil [51]. Some researchers also found that using BIS monitoring did not influence the extubation time and discharge time [49,52]. Our results were similar to those in the above reports; the remifentanil infusion rate and MAP in the anesthesia maintenance period were similar between the two groups, and extubation time and discharge time also showed no differences between the two groups. In addition, the EtSevo in the maintenance period was lower in the E group. This suggests that for anesthesiologists, using EEG-related parameters for monitoring in children may be a beneficial choice.

In 2015, Mehmet et al. found that using the BIS for monitoring anesthesia depth reduced postoperative pain in pediatric patients undergoing dental surgery [53]. A randomized controlled study also found that using the BIS for monitoring anesthesia depth could reduce FLACC scores and NRS scores, even though no statistical significance was found [49]. These were consistent with our trial results, which indicated that monitoring EEG parameters intraoperatively could effectively reduce pain scores within 30 min from extubation. We deemed that this result could support the benefit of using EEG-related parameters for monitoring in pediatric patients.

Since EEG monitoring has been introduced in pediatric anesthesia in 2000 [21], scholars have carried out some studies on the influence of its application on the emergence stage [25,26,52]. An observational study found that in 400 children undergoing dental operations with inhalation anesthesia, the incidence of EA was not related with deeper anesthesia (BIS < 45) [52]. Similarly, in Larsen’s trial, in pediatric patients aged 1–6 years undergoing day case surgery, Narcotrend monitoring could not reduce the rate of EA, which was consistent with our research results [25]. In this study of pediatric patients undergoing hypospadias surgery with sevoflurane anesthesia, maintaining proper anesthesia depth via the comprehensive judgment of the SEF, DSA, and raw EEG caused a decreased incidence of ED as the primary outcome. However, in respect of reducing EA, the incidence of EA in group E was lower than that in the C group, but there were no statistical differences. This study also found that the E group had a shorter duration of deep anesthesia than the C group. Therefore, the primary outcome, combined with the above secondary results, may help anesthesiologists to performed better anesthesia management for pediatric surgery in future clinical work.

There were also some limitations. Firstly, in the pediatric population, brain development gradually improves with growing up; however, the ages of the participants in our trial ranged 2–12 years, which may be a large age span. So, smaller age ranges should be considered in future studies in order to obtain more convincing results. Secondly, due to the limitation on surgery time (>1 h), the sample size was not big enough, so it is necessary to expand the sample size and enrich the type of surgery to make results more universal.

## 5. Conclusions

In conclusion, using EEG-related parameters to guide anesthesia depth could reduce the occurrence of ED and could reduce the dosage of sevoflurane for anesthesia maintenance without increasing the intraoperative analgesics. 

## Figures and Tables

**Table 1:** Baseline characteristics of two groups.

	C Group (*n* = 19)	E Group (*n* = 18)	*p*-Value
Age (years)	4.79 ± 2.92	5.67 ± 2.77	0.285
Heigh (cm)	110.19 ± 24.55	117.25 ± 23.35	0.521
Weight (Kg)	18 (14.0, 25.25)	23 (18, 40)	0.100
ASA			0.832
Ⅰ	16 (89%)	15 (88%)	
Ⅱ	2 (11%)	2 (12%)	
Intubation type			0.479
Endotracheal	7 (37%)	9 (50%)	
Laryngeal	12 (63%)	9 (50%)	
History of surgery	4 (21%)	2(11%)	0.660
Length of anesthesia (min)	85.0 (64.5, 123.5)	83.0 (76.0, 103.0)	0.302

Data are expressed as means ± SDs, medians (interquartile ranges), or numbers of patients (%) as appropriate. ASA, American Society of Anesthesiologists.

**Table 2:** Primary and secondary outcomes in the PACU.

	C Group (*n* = 19)	E Group (*n* = 18)	*p*-Value
ED	7 (36.8%)	1 (5.6%)	0.042 *
EA	15 (78.9%)	11 (61.1%)	0.476
Extubation time (min)	28.7 ± 11.8	28.1 ± 13.5	0.861
Discharge time (min)	83.37 ± 23.61	75.8 ± 25.1	0.679
FLACC scores ^#^	2 (1,3)	0 (0,2)	0.032 *
Adverse events (%)	3 (15.8%)	1 (5.5%)	0.604

Data are expressed as means ± SDs, medians (interquartile ranges), or numbers of patients (%) as appropriate. PACU, post anesthesia care unit; ED, emergence delirium; EA, emergence agitation; FLACC, Faces, Legs, Activity, Cry, and Consolability; * *p* < 0.05; ^#^ highest FLACC scores in the PACU.

**Table 3:** Comparison results between two groups intraoperatively.

	C Group	E Group	*p*-Value
Induction EtSevo (et Vol%)	5.8 ± 1.6	5.6 ± 1.9	0.476
Maintenance EtSevo (et Vol%) *	2.8 ± 0.7	2.3 ± 0.9	0.033
Burst suppression (cases)	3	1	0.791
Cisatracurium (mg)	4.7 ± 2.5	6.1 ± 2.4	0.106
Remifentanil (ug/kg/min)	0.18 ± 0.8	0.19 ± 0.4	0.984
Decrease in MAP (%) ^#^	14.6 ± 7.1	16.2 ± 7.1	0.947

Data are expressed as means ± SDs, end-tidal sevoflurane concentration; MAP, mean arterial pressure; * *p* < 0.05; ^#^ Max decrease in MAP during anesthesia.

**Table 4:** SEF characteristics during anesthesia.

	C Group	E Group	*p*-Value
SEFL < 10 (min)	21.3 ± 10.1	15.8 ± 5.6	0.049 *
SEFR < 10 (min)	20.8 ± 11.4	16.9 ±6.6	0.208
SEFL + SEFR < 10 (min)	17.6 ± 9.2	12.1 ± 4.6	0.028 *
SEFL > 15 (min)	20.4 ± 7.6	19.3 ± 7.9	0.689
SEFR > 15 (min)	22.1 ± 7.5	20.3 ± 8.2	0.524
SEFL + SEFR>15 (min)	18.0 ± 5.9	17.1 ± 7.5	0.690

Data are expressed as means ± SDs. SEF, spectral edge frequency; SEFL, SEF of left brain; SEFR, SEF of right brain; SEFL + SEFR, figure simultaneously representing SEFL and SEFR < 10 or > 15; * *p* < 0.05.

## Data Availability

The data that support the findings of this study are available on request from the corresponding author. The data are not publicly available due to privacy or ethical restrictions.
